# Comparative Endothelialization of the Watchman Plug Device and LACBES Pacifier Occluder after Left Atrial Appendage Closure

**DOI:** 10.31083/j.rcm2512450

**Published:** 2024-12-23

**Authors:** Jing Zhou, Zongqi Zhang, Kandi Zhang, Tiantian Zhang, Qing He, Junfeng Zhang

**Affiliations:** ^1^Department of Cardiology, Shanghai Ninth People's Hospital, Shanghai Jiao Tong University School of Medicine, 200011 Shanghai, China

**Keywords:** nonvalvular atrial fibrillation, left atrial appendage closure, endothelialization, cardiac computed tomography angiography

## Abstract

**Background:**

For patients with nonvalvular atrial fibrillation (NVAF), left atrial appendage closure (LAAC) is an alternative to oral anticoagulants (OACs). However, incomplete device endothelialization (IDE) after LAAC has been linked to device-related thrombus (DRT) and subsequent thromboembolic events. Here, the differences in device endothelialization between the Watchman plug device and the LACBES pacifier occluder after implantation were investigated.

**Methods:**

Of 201 consecutive patients with indications for LAAC, 101 received a Watchman 2.5 device, and 100 received a LACBES occluder. IDE was defined as a residual flow of contrast agent inside the left atrial appendage (LAA) on cardiac computed tomography angiography (CCTA) without peri-device leak (PDL) at the 3-month and 6-month follow-ups.

**Results:**

There were no significant differences in DRT or PDL incidence between the two groups. However, the IDE rate in the absence of PDL was higher in the LACBES group than in the Watchman group at 3 months (42.4% versus 25.8%; *p* = 0.025) and at the 6-month follow-up (24.7% versus 11.2%; *p* = 0.028) as determined by CCTA.

**Conclusions:**

Our findings indicated that the LACBES occluder took longer to complete endothelialization than the Watchman device after successful LAAC therapy. CCTA is a reliable imaging method for assessing the sealing of LAAC devices and confirming complete device endothelialization.

## 1. Introduction

Atrial fibrillation (AF) is the most common tachyarrhythmia, and more than 90% of AF cases are nonvalvular atrial fibrillation (NVAF). Since more than 90% of thrombi in NVAF patients originate from the left atrial appendage (LAA) [1], percutaneous left atrial appendage closure (LAAC) has emerged as a valid therapeutic option for significantly decreasing stroke risk by reducing the risk of bleeding complications associated with long-term oral anticoagulant (OAC) therapy [2].

Currently, two primary types of LAAC devices are used in clinical practice: the plug occluder represented by Watchman, and the pacifier occluder represented by Amulet and LACBES. The Watchman 2.5 device, widely utilized worldwide, features a 10-strut nitinol frame coated with a polyethylene terephthalate membrane on its atrial surface to enhance device endothelialization (**Supplementary Table 1**) [3]. The LACBES occluder is a new dual-seal LAAC device constructed from a nitinol mesh, consisting of a distal anchoring lobe and a proximal sealing disc (**Supplementary Table 1**) [4, 5].

Similar to other implanted cardiac devices, there exists a specific timeframe during the endothelialization period in which the foreign metallic material of an LAAC device is exposed to blood on the atrial side, which can activate the coagulation cascade. Although this step is essential for the healing process related to the implantation of a LAAC device, it might have the potential to cause device related thrombus (DRT) on the nonendothelialized surface, possibly resulting in thromboembolic events. Previous meta-analyses have shown that pacifier occluders are associated with increased rates of major procedure-related complications, while the plug occluders are linked to higher incidences of DRT and peri-device leakage (PDL) >5 mm [6, 7]. Studies have shown that the degree of endothelialization on the occluder surface after LAAC is a key factor affecting DRT formation [8, 9]. Animal studies indicate that approximately 45 days after LAAC, the surface of the plug occluder is covered by newly formed endothelial cells, while complete endothelialization takes longer with the pacifier occluder [4, 10]. In fact, delayed endothelialization of the device after implantation is frequently described in humans [11, 12]. However, few studies have directly compared the Watchman and LACBES devices in terms of complete device endothelialization (CDE) after LAAC. Therefore, the purpose of this research was to investigate the differences in device endothelialization between the Watchman plug device and the LACBES pacifier occluder after implantation.

## 2. Methods

### 2.1 Study Population and Design

A total of 201 NVAF patients who underwent successful LAAC treatment and completed a 6-month follow-up at the Department of Cardiology at Shanghai Ninth People’s Hospital (Shanghai, China) between June 2021 and September 2023 were included in this retrospective observational single-center study (**Supplementary Fig. 1**). The procedures were performed using either the Watchman 2.5 device or the LACBES occluder. The participants in the study were required to meet the following criteria: were at least 18 years old, had a CHA_2_DS_2_-VASc score greater than 2, and either had a HAS-BLED score of 2 or higher or refused oral anticoagulant therapy. Patients with terminal illnesses and a life expectancy of less than one year, as well as those with echocardiographic evidence of thrombus in the left atrium or LAA, were excluded from LAAC therapy. The data collected included patient demographics, clinical characteristics, procedural data and follow-up clinical events. This research complied with the Declaration of Helsinki guidelines and was approved by our institution’s ethics committee. All patients were fully informed about the procedure and provided written informed consent.

### 2.2 LAAC Procedure

Cardiac computed tomography angiography (CCTA) was performed before intervention to determine the vacuity and anatomy of the LAA and ensure that the closure device was compatible with it [13]. Fluoroscopy and transesophageal echocardiography (TEE) guided the LAAC under general anesthesia. A Philips CX50 color Doppler echocardiography system (Philips Ultrasound, Bothell, WA, USA) was used for the TEE examinations according to the manufacturer’s guidelines. The selection of the LAAC occluder in each patient was based on the operator’s discretion. During implantation, guidance of the transseptal puncture, evaluation of the correct position in the LAA, assessment of the amount of protrusion, determination of PDL (residual visible continuity of contrast between LA and LAA along the side of the device), tug-testing to ensure a stable position, and evaluation of spontaneous echocontrast, thrombi and pericardial effusion were performed using TEE as described previously [14]. Under continuous pressure guidance, a transseptal puncture was performed as low and posteriorly as possible based on TEE. All patients received intravenous heparin 100 IU/kg after the atrial septum was punctured to maintain an activated clotting time (ACT) greater than 250 seconds. In the context of LAAC angiograms and TEE, the optimal device size was determined. All device implantations fulfilled the Position-Anchoring-Size-Seal (PASS) criteria for the Watchman device or the Proper Position-Absolute Anchor-Separate Seal-Typical Tire (PAST) criteria for the LACBES device prior to device release. Successful LAA closure was confirmed by TEE and fluoroscopy, which was defined as the absence of PDL or a PDL ≤5 mm [14].

### 2.3 Postprocedure Management

After the LAAC procedure, the patients were observed overnight and discharged the next day following the exclusion of patients with significant pericardial effusion/tamponade, major bleeding related to the procedure, or other severe periprocedural complications. During the first 3 months post-procedure, the recommended antithrombotic regimen comprised OACs for the Watchman device group and either OACs or dual antiplatelet therapy (DAPT) for the LACBES occluder group. However, the specific drug regimen after implantation was determined by the operator based on factors such as bleeding risk, stroke risk, and post-implantation echocardiography. At the 3-month visit, OACs were discontinued, and the patients were given DAPT if TEE showed adequate closure of the LAA with no apparent residual PDL (≤5 mm in width) or DRT. DRT was identified as a clot that formed on the atrial surface of the device during or after its implantation, as previously described [14, 15]. At the 6-month visit, clopidogrel was discontinued; aspirin was continued indefinitely. If inadequate peri-device flow was obtained or DRT was detected, anticoagulation therapy was restarted with OACs until an adequate seal or complete disappearance of the thrombus was confirmed by a repeat TEE exam.

### 2.4 Follow‑up TEE Imaging

TEE was performed at the 3-month follow-up to evaluate the correct device position and the degree of PDL, with an additional TEE scheduled at the 6-month visit if a residual PDL >5 mm or DRT was detected. Both two-dimensional (2D) and three-dimensional (3D) TEE were performed according to standard guidelines, and views of the LAA were obtained at 0°, 45°, 90°, and 135° at the mid-esophageal level. The PDL Doppler imaging was performed in each view by an echocardiographer blinded to the procedure and CCTA results and trained in device follow-up.

### 2.5 Follow‑up CCTA Imaging

After a successful LAAC procedure, all patients were required to undergo CCTA at least twice: at 3 months and 6 months. The CCTA protocol has been described previously [16]. In brief, contrast-enhanced electrocardiogram (ECG)-gated CCTA imaging was performed on a 64-slice SOMATOM definition flash dual-source computed tomography (CT) scanner (Siemens, Forchheim, Germany) with multiphasic acquisition in the arterial and venous phases. The temporal resolution was 330 ms, and the detector collimation was 64 × 0.6 mm. A 100 mL bolus of iodixanol was injected through the elbow vein at a rate of 5 mL/s as the contrast agent. Following injection, the delayed scan (venous phase) was executed 60 seconds after the beginning of the standard scan to allow contrast equilibration within the blood pool. Incomplete endothelialization of LAAC devices was assessed by measuring the Hounsfield unit (HU) in the left atrium (LA) and the LAA as described previously [16, 17]. An LAA density of <100 HU and <25% of that of the LA was considered complete occlusion, as previously suggested [18, 19]. Any LAA exhibiting a local density ≥100 HU or ≤25% of that of the LA was considered patent, indicating incomplete device endothelialization (IDE).

### 2.6 Statistical Analysis

Standard descriptive statistical methods were used: absolute and relative frequencies were reported for categorical data, and the median (interquartile range, IQR) or mean ± standard deviation (SD) were reported for continuous data. Continuous variables were analyzed for a normal distribution using the Shapiro-Wilk test. To assess the differences between two continuous variables, the Student’s *t*-test (for normally distributed values) or the Mann-Whitney U test (for nonnormally distributed values) was used. Categorical variables were assessed using Fisher’s exact test. Statistical analysis was performed with GraphPad Prism 9.0 software (GraphPad Software, San Diego, CA, USA). A two-sided *p* value <0.05 was considered to indicate statistical significance.

## 3. Results

### 3.1 Baseline Demographic and Clinical Characteristics

Between June 2021 and September 2023, LAAC procedures were performed in 201 patients: the Watchman device was implanted in 101 patients, while the LACBES device was utilized in 100 patients. The two groups exhibited almost no significant differences, except for the average device size. The mean patient age was 72.9 years, and 79 (39.3%) patients were women. The mean CHA_2_DS_2_-VASc score was 4, and the mean HAS-BLED score was 2. A history of stroke or transient ischemic attack (TIA) was present in more than 25% of patients. At the 3-month follow-up visit, most (91.1%) patients who received the Watchman device were on OACs. Sixty-seven percent of patients with the LACBES occluder were on OACs, and 28% were on DAPT. However, antithrombotic therapy was not significantly different between the two groups at the 6-month follow-up visit. The baseline demographic and clinical characteristics are summarized in Table 1. The LAA shape based on preprocedural CCTA analysis is given in** Supplementary Table 2**.

**Table 1.** **Baseline demographic and clinical characteristics**.

Patient characteristics		Watchman (n = 101)	LACBES (n = 100)	*p* value
Age (year), mean (SD)		72.3 ± 8.3	73.4 ± 6.8	0.326
Male gender, n (%)		63 (62.4)	59 (59.0)	0.666
BMI (kg/m^2^), median (IQR)		25.7 (23.9–27.3)	24.9 (23.0–26.8)	0.066
Paroxysmal AF, n (%)		60 (59.4)	54 (54.0)	0.478
Hypertension, n (%)		76 (75.3)	77 (77.0)	0.869
Diabetes, n (%)		30 (29.7)	33 (33.0)	0.650
Previous stroke/TIA, n (%)		28 (27.7)	28 (28.0)	>0.999
CAD, n (%)		35 (34.7)	40 (40.0)	0.468
CHF, n (%)		34 (33.7)	28 (28.0)	0.382
CKD, n (%)		22 (21.8)	16 (16.0)	0.368
Liver dysfunction, n (%)		4 (4.0)	7 (7.0)	0.373
Prior major bleeding or predisposition to bleeding, n (%)		12 (11.9)	8 (8.0)	0.481
CHA_2_DS_2_-VASc score, median (IQR)		4 (2–5)	4 (3–5)	0.679
HAS-BLED score, median (IQR)		2 (2–3)	2 (2–3)	0.286
EF (%), median (IQR)		58 (55–62)	59 (55–64)	0.644
GFR (mL/min), mean (SD)		75.3 ± 20.4	74.6 ± 18.6	0.820
LA dimension (mm), median (IQR)		43 (40–47)	43 (38–48)	0.664
Maximum diameter of LAA orifice (TEE mm), mean (SD)		22.6 ± 2.9	22.8 ± 4.0	0.707
Antithrombotic therapy at 3 months				<0.0001
	OACs, n (%)	92 (91.1)	67 (67.0)	
	DAPT, n (%)	0 (0.0)	28 (28.0)	
	Other, n (%)	9 (8.9)	5 (5.0)	
Antithrombotic therapy at 6 months				0.547
	OACs, n (%)	14 (13.9)	12 (12.0)	
	DAPT, n (%)	81 (80.2)	78 (78.0)	
	Other, n (%)	6 (5.9)	10 (10.0)	

Abbreviations: AF, atrial fibrillation; BMI, body mass index; CAD, coronary artery disease; CHF, chronic heart failure; CKD, chronic kidney disease; DAPT, dual antiplatelet therapy; EF, ejection fraction; GFR, glomerular filtration rate; IQR, interquartile range; LA, left atrium; LAA, left atrial appendage; OACs, oral anticoagulations; SD, standard deviation; TEE, transesophageal echocardiography; TIA, transient ischemic attack.

### 3.2 Periprocedural and 6-Month Follow-up Clinical Events after LAAC

The perioperative period and follow-up clinical events at 6 months after LAAC are presented in Table 2. The implantation procedure was successful in all patients in both groups, without any observed periprocedural complications, such as cardiac tamponade, stroke or TIA, or device embolization. There were no statistically significant differences in peripheral vascular complications between the Watchman group (4.0%) and the LACBES group (5.0%). No occurrences of stroke or TIA, other systemic embolism, peripheral vascular complications or deaths were observed in either group during the 6-month follow-up after hospital discharge. Bleeding complications occurred at comparable rates between the two groups (2.9% versus 2.0%).

**Table 2.** **Periprocedural and 6-month follow-up clinical events after LAAC**.

		Watchman (n = 101)	LACBES (n = 100)	*p* value
Periprocedural clinical events				
	Failure of implantation, n (%)	0 (0.0)	0 (0.0)	/
	Cardiac tamponade, n (%)	0 (0.0)	0 (0.0)	/
	Stroke or TIA, n (%)	0 (0.0)	0 (0.0)	/
	Device embolization, n (%)	0 (0.0)	0 (0.0)	/
	Peripheral vascular complication, n (%)	4 (4.0)	5 (5.0)	0.748
6-month follow-up clinical events				
	Stroke or TIA, n (%)	0 (0.0)	0 (0.0)	/
	Other systemic embolism, n (%)	0 (0.0)	0 (0.0)	/
	Bleeding complication, n (%)	3 (2.9)	2 (2.0)	>0.999
	Peripheral vascular complication, n (%)	0 (0.0)	0 (0.0)	/
	6-month mortality, n (%)	0 (0.0)	0 (0.0)	/

Abbreviations: LAAC, left atrial appendage closure; TIA, transient ischemic attack.

### 3.3 Device Endothelialization on Follow-up TEE and CCTA

**Fig. 1.**
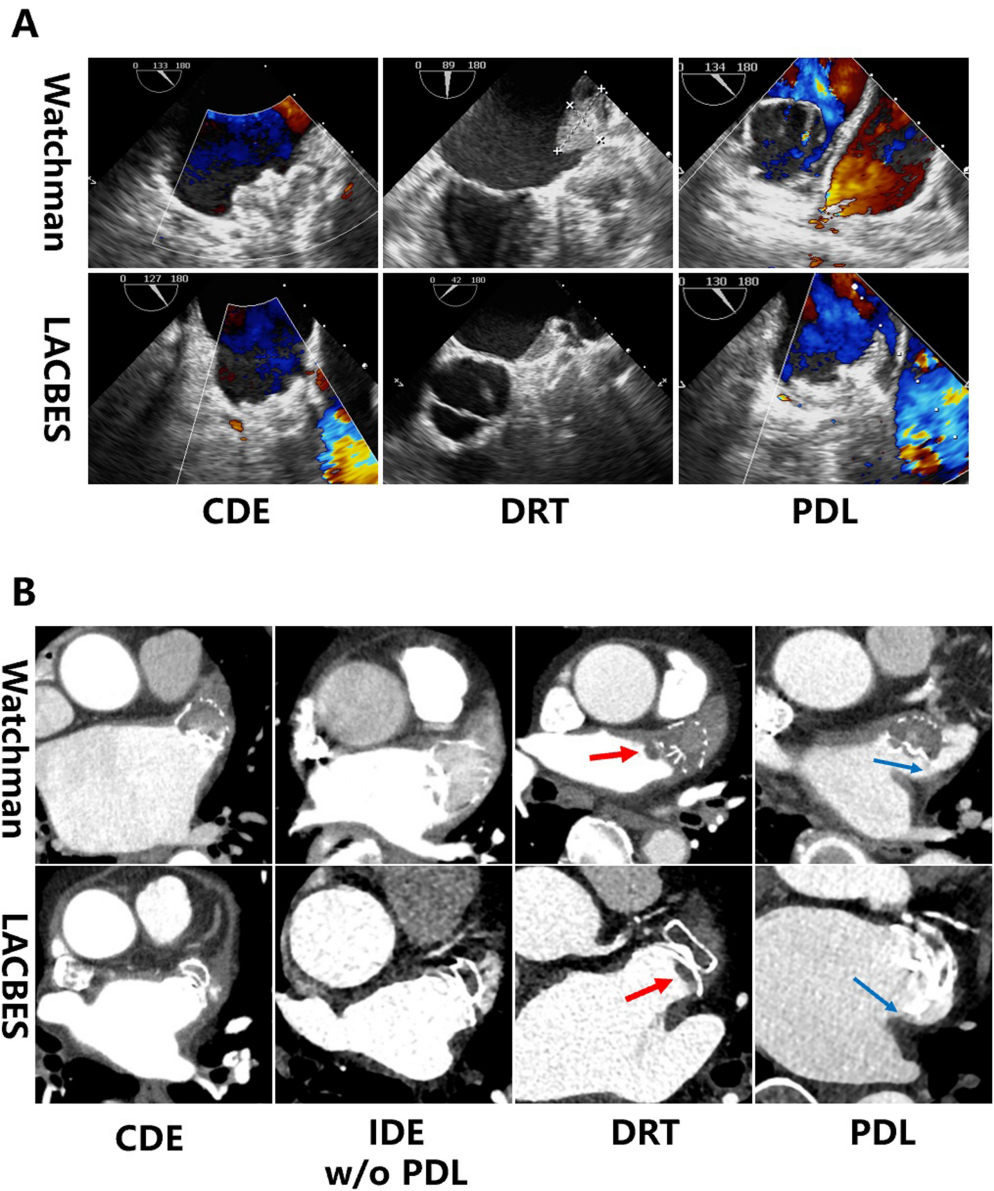
**Analysis of device implantation on follow-up TEE and CCTA images**. (A) shows representative examples of CDE, DRT and PDL in patients with Watchman 2.5 or LACBES devices as determined by TEE. (B) shows representative examples of CDE, IDE w/o PDL, DRT (red arrow) and PDL (blue arrow) in patients with Watchman or LACBES devices as determined by CCTA. Abbreviations: CCTA, cardiac computed tomography angiography; CDE, complete device endothelialization; DRT, device related thrombus; IDE, incomplete device endothelialization; PDL, peri-device leak; TEE, transesophageal echocardiography; w/o, without.

**Fig. 2.**
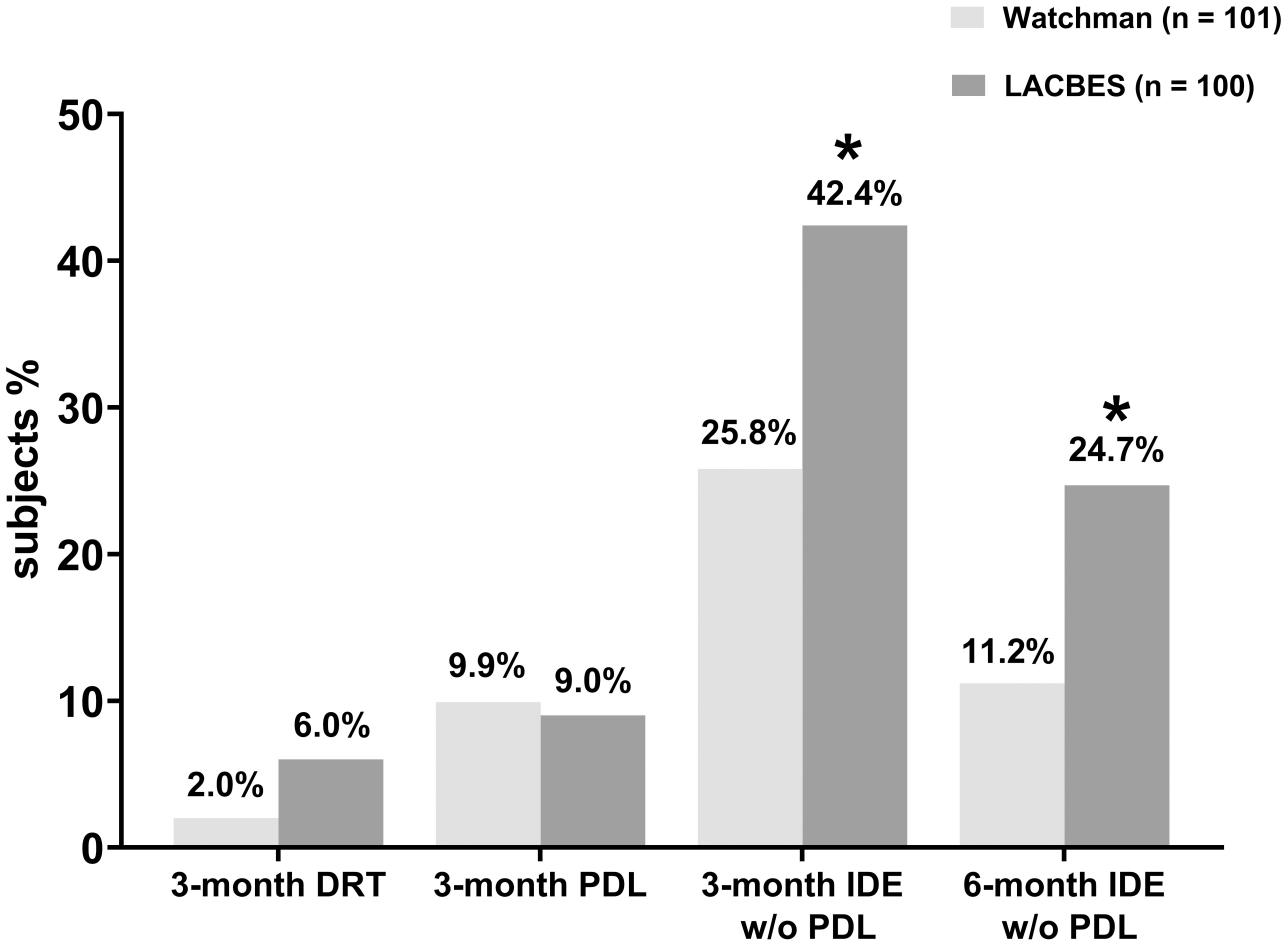
**Comparative analysis of DRT, PDL and device endothelialization during postprocedure follow-up**. The rates of DRT, PDL, and IDE without PDL were compared between the Watchman 2.5 group and the LACBES group, respectively. * *p* < 0.05 *vs* the Watchman group. Abbreviations: DRT, device related thrombus; IDE, incomplete device endothelialization; PDL, peri-device leak; w/o, without.

TEE was performed 3 months after LAAC (a representative example is shown in Fig. 1). DRT was detected in 2 patients with the Watchman device and in 6 patients with the LACBES occluder. The rate of DRT in the Watchman group was marginally lower than that in the LACBES group, although this difference did not reach statistical significance (2.0% versus 6.0%, *p* = 0.170). Furthermore, CCTA was performed at 3 months and 6 months after LAAC (a representative example is shown in Fig. 1). The rate of PDL occurrence was also comparable between the two groups at 3 months according to TEE and CCTA, with rates of 9.9% and 9.0%, respectively. CCTA at the 3-month follow-up revealed contrast leakage in the LAA without PDL in 23/91 patients (25.8%) in the Watchman group, suggesting the occurrence of IDE. In contrast, 36/85 patients (42.4%) in the LACBES group had residual flow inside the LAA in the absence of PDL, which was significantly higher than that in the Watchman group (*p* = 0.025). Moreover, the incidence of IDE without PDL in the LACBES group remained significantly greater than that in the Watchman group at the 6-month follow-up (24.7% versus 11.2%; *p* = 0.028), as determined by CCTA. The aforementioned findings are summarized in Fig. 2.

## 4. Discussion

In this study, we combined TEE and CCTA for follow-up of LAAC to investigate the differences in device endothelialization between the Watchman plug device and the LACBES pacifier occluder. TEE is more suitable for observing the PDL and less sensitive to IDE, while CCTA is more objective and accurate for evaluating endothelialization on the device surface [16]. We observed that the rate of DRT was not significantly different between the two groups, as determined by TEE and CCTA. In addition, the PDL rate was also comparable between the two groups at 3 months. However, the IDE rate in the absence of PDL was considerably greater in the LACBES group compared to the Watchman group at the 3-month follow-up. Moreover, the incidence of IDE without PDL in the LACBES group remained significantly higher than that in the Watchman group at the 6-month follow-up, as determined by CCTA.

Device endothelialization is considered the most effective factor in reducing DRT. In theory, a complete endothelial layer covering the interface between the device and blood provides a smooth and clot-resistant surface. Initial animal studies have suggested that near-complete endothelialization can be achieved within 45 days after LAAC [10]. However, these studies involved healthy young animals with intracardiac anatomy and physiology distinct from those observed in elderly patients with NVAF. The Watchman device has been shown to remain porous 6 weeks after implantation in a substantial percentage of patients, suggesting delayed endothelialization of the device from a small-sample study. Specifically, complete occlusion was observed in only 18 out of 46 patients (41%) at the 45-day follow-up [11]. In a recent study, CCTA images revealed IDE without a visible PDL in 7 out of 51 AF patients (14%) post Watchman LAAC at 6 months postprocedure [20]. The same CCTA method was used in our study to assess IDE, yielding comparable findings. The results from a LACBES LAAC prospective study showed that the percentage of CDE was 39.7%, and the incidence of DRT was 4.2%, as detected by CCTA at 3 months after implantation [21]. Our findings showed a greater proportion of CDE and a similar occurrence of DRT in those who received the LACBES device compared to the above study. Moreover, the higher ratio of IDE observed with the LACBES occluder compared to the Watchman device in our study may be attributed to differences in conceptual device design as well as device size. The SWISS APERO trial revealed a similar percentage of patients with a patent LAA between the Amulet pacifier occluder (67.6%) and the Watchman 2.5 or FLX plug devices (70%) in patients who underwent CCTA at 45 days after LAAC [14]. The Amulet-IDE trial revealed a higher incidence of PDL with the Watchman 2.5 than with the Amulet device, as assessed by TEE at 45 days. However, CCTA was not employed in this study to compare the occurrence of IDE between the Amulet and Watchman 2.5 devices [22]. Due to the limited follow-up period for the IDE and PDL compared between plug- and pacifier-type devices in these two studies, it is difficult to draw valuable conclusions regarding device endothelialization. The incidence of IDE in our cohort was relatively low because most previous studies were conducted within a timeframe shorter than 6 months, unlike our study.

Recently, complete LAA occlusion was significantly greater with Watchman FLX, the new generation of Watchman device, compared to the Amulet device at 2-month CCTA follow-up [23]. The higher rate of complete occlusion with the Watchman FLX device may indicate enhanced device endothelialization. Furthermore, fluoropolymer-coated Watchman FLX (FP-WM), the latest LAAC product, was shown to have less thrombogenicity and superior endothelial coverage than conventional uncoated Watchman FLX in a canine model [24]. This novel device design may accelerate endothelialization and lower the risk of DRT. Another study used an endothelium-mimicking nanomatrix on a LAAC device membrane to deliver nitric oxide for improved endothelialization, which could reduce the need for anticoagulation treatments for patients [25].

Current imaging techniques for the detection of LAAC endothelialization include CCTA, TEE, and intracardiac ultrasound. However, there is still a need for a more precise method to evaluate device endothelialization. Recently, CCTA combined with the UNet neural network model (deep learning) has facilitated the quantitative assessment of Watchman device endothelialization. The endothelialization ratio was automatically determined when the investigators identified hypoattenuated thickening (HAT) regions, and a HAT/LA attenuation ratio higher than 0.2 was considered to indicate endothelialization [26]. However, the clinical significance of this method warrants further scrutiny in the various types of LAAC devices.

## 5. Limitations

Here, we acknowledge the inherent limitations of our study. Firstly, the study was limited by a small sample size and a retrospective analysis conducted at a single center, which may introduce some degree of selection bias that cannot be completely eliminated. Furthermore, there are currently no established guidelines or expert consensuses regarding the selection of the optimal LAAC device for patients with varying LAA anatomical morphologies. Hence, reducing device selection bias during the LAAC procedure may present challenges. Secondly, the limited number of patients and the short duration of clinical follow-up restricted our ability to fully assess the long-term outcomes of patients with IDE and to determine the superiority of one device over another. Thirdly, the association between IDE and embolic stroke, as well as its impact on patient outcomes, remains uncertain.

## 6. Conclusions

In conclusion, our findings indicated that the LACBES occluder took longer to complete endothelialization than the Watchman device after a successful LAAC procedure during the 6-months of follow-up. The incidences of DRT and PDL were similar between the two groups. The differences in conceptual device design complicate the interpretation of these results, highlighting the need for further randomized head-to-head studies with clinical outcomes. Additionally, CCTA is a reliable imaging method for assessing the sealing of LAAC devices and confirming CDE.

## Data Availability

The data sets generated and/or analysed during the current study are available from the corresponding author upon reasonable request.

## Supplementary Material

Supplementary material associated with this article can be found, in the online version, at https://doi.org/10.31083/j.rcm2512450.

## References

[1] Klein AL, Grimm RA, Murray RD, Apperson-Hansen C, Asinger RW, Black IW (2001). Use of transesophageal echocardiography to guide cardioversion in patients with atrial fibrillation. *The New England Journal of Medicine*.

[2] Joglar JA, Chung MK, Armbruster AL, Benjamin EJ, Chyou JY, Cronin EM (2024). 2023 ACC/AHA/ACCP/HRS Guideline for the Diagnosis and Management of Atrial Fibrillation: A Report of the American College of Cardiology/American Heart Association Joint Committee on Clinical Practice Guidelines. *Circulation*.

[3] Maksym J, Grabowski M, Mazurek T (2024). Percutaneous left atrial appendage closure with the Watchman device: a systematic review. *Advances in Interventional Cardiology*.

[4] Tang X, Zhang Z, Wang F, Bai Y, Xu X, Huang X (2017). Percutaneous Left Atrial Appendage Closure With LACBES® Occluder - A Preclinical Feasibility Study. *Circulation Journal: Official Journal of the Japanese Circulation Society*.

[5] Bai Y, Tang X, Xu X, Zhao X, Xu Y, Chen W (2022). A newly designed disk-lobe occluder with isogenous barbs for left atrial appendage closure: Initial multicenter experience. *Frontiers in Cardiovascular Medicine*.

[6] Bing S, Chen RR (2023). Clinical efficacy and safety comparison of Watchman device versus ACP/Amulet device for percutaneous left atrial appendage closure in patients with nonvalvular atrial fibrillation: A study-level meta-analysis of clinical trials. *Clinical Cardiology*.

[7] Qiao J, Zhang B, Wang J, Pan L, Cheng T, Wang Y (2022). Comparison between Amplatzer and Watchman Left Atrial Appendage Closure Devices for Stroke Prevention in Atrial Fibrillation: A Systematic Review and Meta-Analysis. *Cardiology*.

[8] Dukkipati SR, Kar S, Holmes DR, Doshi SK, Swarup V, Gibson DN (2018). Device-Related Thrombus After Left Atrial Appendage Closure: Incidence, Predictors, and Outcomes. *Circulation*.

[9] Fauchier L, Cinaud A, Brigadeau F, Lepillier A, Pierre B, Abbey S (2018). Device-Related Thrombosis After Percutaneous Left Atrial Appendage Occlusion for Atrial Fibrillation. *Journal of the American College of Cardiology*.

[10] Schwartz RS, Holmes DR, Van Tassel RA, Hauser R, Henry TD, Mooney M (2010). Left atrial appendage obliteration: mechanisms of healing and intracardiac integration. *JACC. Cardiovascular Interventions*.

[11] Sivasambu B, Arbab-Zadeh A, Hays A, Calkins H, Berger RD (2019). Delayed endothelialization of watchman device identified with cardiac CT. *Journal of Cardiovascular Electrophysiology*.

[12] Lindner S, Behnes M, Wenke A, Sartorius B, Akin M, Mashayekhi K (2021). Incomplete neo-endothelialization of left atrial appendage closure devices is frequent after 6 months: a pilot imaging study. *The International Journal of Cardiovascular Imaging*.

[13] Zhang K, Zhou J, Zhang T, Zhang Z, Jin S, He Q (2022). Comparison of multiple imaging modalities for measuring orifice diameter and selecting occluder size in patients undergoing left atrial appendage closure. *Clinical Cardiology*.

[14] Galea R, De Marco F, Meneveau N, Aminian A, Anselme F, Gräni C (2022). Amulet or Watchman Device for Percutaneous Left Atrial Appendage Closure: Primary Results of the SWISS-APERO Randomized Clinical Trial. *Circulation*.

[15] Korsholm K, Jensen JM, Nørgaard BL, Nielsen-Kudsk JE (2019). Detection of Device-Related Thrombosis Following Left Atrial Appendage Occlusion: A Comparison Between Cardiac Computed Tomography and Transesophageal Echocardiography. *Circulation. Cardiovascular Interventions*.

[16] Qamar SR, Jalal S, Nicolaou S, Tsang M, Gilhofer T, Saw J (2019). Comparison of cardiac computed tomography angiography and transoesophageal echocardiography for device surveillance after left atrial appendage closure. *EuroIntervention: Journal of EuroPCR in Collaboration with the Working Group on Interventional Cardiology of the European Society of Cardiology*.

[17] Galea R, Gräni C (2021). Device neo-endothelialization after left atrial appendage closure: the role of cardiac computed tomography angiography. *The International Journal of Cardiovascular Imaging*.

[18] Saw J, Fahmy P, DeJong P, Lempereur M, Spencer R, Tsang M (2015). Cardiac CT angiography for device surveillance after endovascular left atrial appendage closure. *European Heart Journal. Cardiovascular Imaging*.

[19] Cochet H, Iriart X, Sridi S, Camaioni C, Corneloup O, Montaudon M (2018). Left atrial appendage patency and device-related thrombus after percutaneous left atrial appendage occlusion: a computed tomography study. *European Heart Journal. Cardiovascular Imaging*.

[20] Zhao MZ, Chi RM, Yu Y, Wang QS, Sun J, Li W (2021). Value of detecting peri-device leak and incomplete endothelialization by cardiac CT angiography in atrial fibrillation patients post Watchman LAAC combined with radiofrequency ablation. *Journal of Cardiovascular Electrophysiology*.

[21] Yao PC, Fei ZT, Chen M, Mo BF, Zhang R, Yang YL (2024). Incidence, impact and predictors of residual device patency after left atrial appendage closure with the LACbes device. *International Journal of Cardiology*.

[22] Lakkireddy D, Thaler D, Ellis CR, Swarup V, Sondergaard L, Carroll J (2021). Amplatzer Amulet Left Atrial Appendage Occluder Versus Watchman Device for Stroke Prophylaxis (Amulet IDE): A Randomized, Controlled Trial. *Circulation*.

[23] Korsholm K, Kramer A, Andersen A, Saw J, Nørgaard BL, Jensen JM (2023). Left atrial appendage sealing performance of the Amplatzer Amulet and Watchman FLX device. *Journal of Interventional Cardiac Electrophysiology: an International Journal of Arrhythmias and Pacing*.

[24] Saliba WI, Kawai K, Sato Y, Kopesky E, Cheng Q, Ghosh SKB (2023). Enhanced Thromboresistance and Endothelialization of a Novel Fluoropolymer-Coated Left Atrial Appendage Closure Device. *JACC. Clinical Electrophysiology*.

[25] Hwang PTJ, Sherwood JA, Millican RC, Bobba PS, Lynd TO, Garner JN (2021). Endothelium-Mimicking Nanomatrix Coating to Enhance Endothelialization after Left Atrial Appendage Closure Device Implantation. *ACS Applied Bio Materials*.

[26] Chen T, Lu X, Wang X, Chen Q, Zhao R, Zhang W (2024). Peri-device leakage and delayed endothelialization of the Watchman device: a computed tomography study. *European Radiology*.

