# Supplementary material for: Comparative Endothelialization of the Watchman Plug Device and LACBES Pacifier Occluder after Left Atrial Appendage Closure
Source: Rev Cardiovasc Med. 2024 Dec 23;25(12):450. doi: 10.31083/j.rcm2512450 (PMC11683694; doi:10.31083/j.rcm2512450)
Supplement: Supplementary file 1 [file 2153-8174-25-12-450-s1.docx]

**Supplemental Material**

**
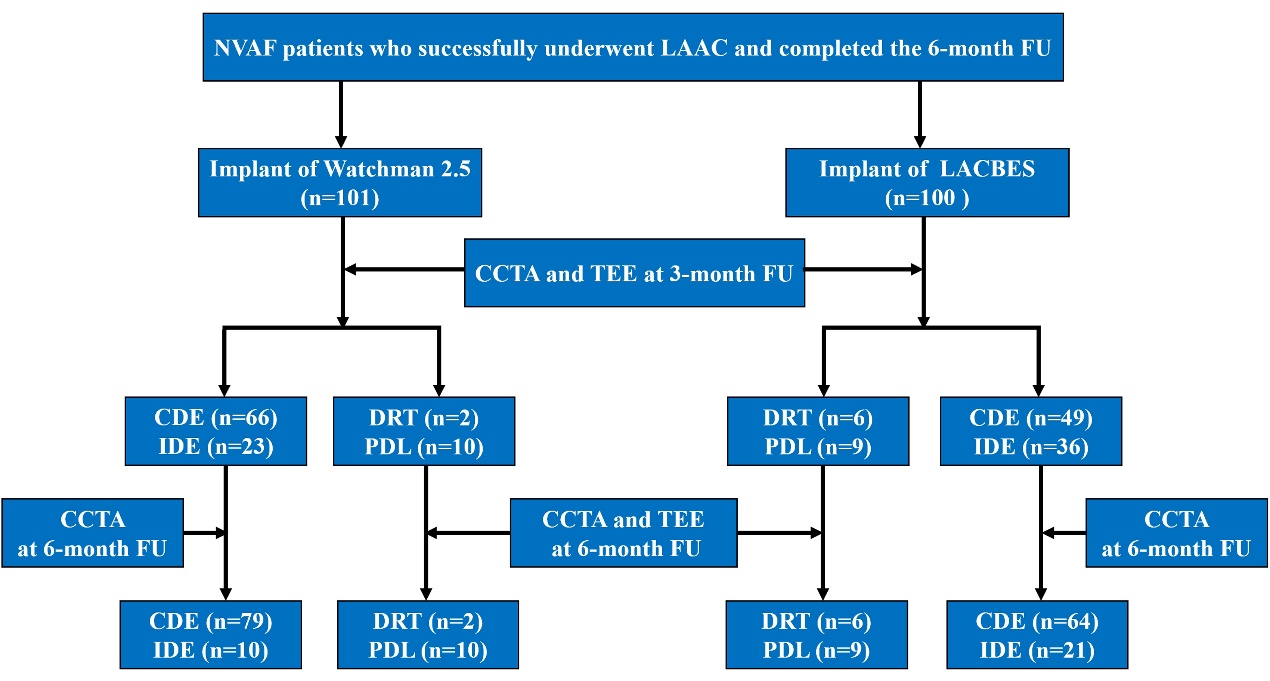
**

**Supplementary Fig. 1. Patient flow chart**

Abbreviations: CCTA: cardiac computed tomography angiography; CDE: complete device endothelialization; DRT: device related thrombus; FU: follow-up; IDE: incomplete device endothelialization; PDL: peri-device leak; TEE: transesophageal echocardiography.

**Supplementary Table 1. Comparison of Devices**

|  | Watchman | LACBES |
| --- | --- | --- |
| Manufacturer | Boston Scientific, USA | Push Medical, China |
| structure of the device | 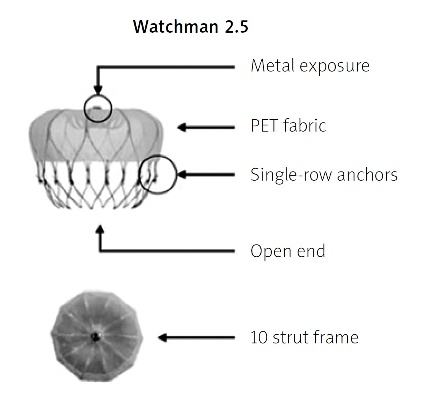 | 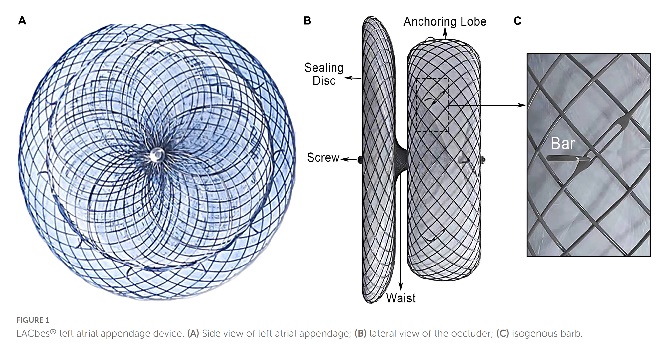 |
| Mechanism of action | Closes off distal body of  left atrial appendage | Lobe of the device placed within the left atrial appendage and the disc sealing the orifice |
| Sizes (diameter) | 21, 24, 27, 30, and 33 mm | 16, 18, 20, 22, 24, 26, 28, 30, 32, and 34 mm |

**References:**

1.Maksym J, Grabowski M, Mazurek T. Percutaneous left atrial appendage closure with the Watchman device: a systematic review. Postepy Kardiol Interwencyjnej. 2024; 20: 18-29.

2.Bai Y, Tang X, Xu X, Zhao X, Xu Y, Chen W, *et al*. A newly designed disk-lobe occluder with isogenous barbs for left atrial appendage closure: Initial multicenter experience. Front Cardiovasc Med. 2022; 9: 974994.

**Supplementary Table 2. The shape of the LAA**

| The shape of the LAA | Watchman (n = 101) | LACBES (n = 100) | *P* value |
| --- | --- | --- | --- |
| Cactus, n (%) | 4 (4.0) | 17 (17.0) | 0.003 |
| Cauliflower, n (%) | 64 (63.4) | 59 (59.0) | 0.564 |
| Chicken wing, n (%) | 25 (24.8) | 17 (17.0) | 0.225 |
| Windsock, n (%) | 8 (7.9) | 7 (7.0) | >0.999 |

**Supplementary Table 3. The incidence of IDE was comparable between patients receiving OACs and DAPT at 3 months and 6 months after LAAC**

| Group | Antithrombotic therapy | IDE w/o PDL | CDE w/o PDL | *P* value |
| --- | --- | --- | --- | --- |
| Watchman group at 3 months | OACs, n (%) | 18 (22.5) | 62 (77.5) | / |
|  | DAPT, n (%) | 0 (0.0) | 0 (0.0) | / |
|  | Other, n (%) | 5 (55.6) | 4 (44.4) | / |
| LACBES group at 3 months | OACs, n (%) | 23 (39.7) | 35 (60.3) | 0.455 |
|  | DAPT, n (%) | 12 (52.2) | 11 (47.8) | / |
|  | Other, n (%) | 1 (25.0) | 3 (75.0) | / |
| Watchman group at 6 months | OACs, n (%) | 2 (20.0) | 8 (80.0) | 0.499 |
|  | DAPT, n (%) | 7 (9.46) | 67 (90.5) | / |
|  | Other, n (%) | 1 (20.0) | 4 (80.0) | / |
| LACBES group at 6 months | OACs, n (%) | 2 (22.2) | 7 (77.8) | 0.962 |
|  | DAPT, n (%) | 18 (26.5) | 50 (73.5) | / |
|  | Other, n (%) | 2 (25.0) | 6 (75.0) | / |

Abbreviations: CDE: complete device endothelialization; DAPT: dual antiplatelet therapy; IDE: incomplete device endothelialization; OAC: oral anticoagulation; PDL: peri-device leak; w/o: without.
